# Clinical value of serum complement component 1q levels in the prognostic analysis of aneurysmal subarachnoid hemorrhage: a prospective cohort study

**DOI:** 10.3389/fneur.2024.1341731

**Published:** 2024-01-31

**Authors:** Linjie Wang, Haotian Zhou, Wenhao Zheng, Heng Wang, Zheng Wang, Xiaoqiao Dong, Quan Du

**Affiliations:** ^1^The Fourth School of Clinical Medicine, Zhejiang Chinese Medical University, Hangzhou, China; ^2^Department of Neurosurgery, Affiliated Hangzhou First People’s Hospital, School of Medicine, Westlake University, Hangzhou, China

**Keywords:** aneurysmal subarachnoid hemorrhage, complement component 1q, prognosis, delayed cerebral ischemia, biomarker

## Abstract

**Objective:**

To analyze the relationship between serum complement component 1q (C1q) levels and functional prognosis in patients with aneurysmal subarachnoid hemorrhage (aSAH), and to reveal its clinical value.

**Methods:**

In this prospective cohort study, we collected clinical data of aSAH patients admitted to the Department of Neurosurgery, Hangzhou First People’s Hospital from January 2020 to October 2022. Parameters such as serum C1q levels, Hunt-Hess grade, modified Fisher grade, and the modified Rankin scale (mRS) at 3 months were included for evaluation. Patients were grouped based on the occurrence of delayed cerebral ischemia (DCI). Spearman rank correlation test and Kruskal-Wallis rank sum test were used to analyze the correlation between serum C1q levels, disease severity, and prognosis. Potential risk factors affecting prognosis and the occurrence of DCI were screened through Independent sample *t*-test or Mann–Whitney *U* test. Variables with significant differences (*p* < 0.05) were incorporated into a logistic regression model to identify independent risk factors affecting prognosis and DCI occurrence. Serum C1q levels were plotted as a ROC curve for predicting prognosis and DCI, and the area under the curve was calculated.

**Results:**

A total of 107 aSAH patients were analyzed. Serum C1q levels positively correlated with Hunt-Hess grade, modified Fisher grade and mRS (all *p* < 0.001). Significant differences were observed in C1q levels among different Hunt-Hess grade, mFisher grade and mRS (all *p* < 0.001). Notably, higher serum C1q levels were seen in the poor prognosis group and DCI group, and correlated with worse prognosis (*OR* = 36.927, 95%*CI* 2.003–680.711, *p* = 0.015), and an increased risk for DCI (*OR* = 17.334, 95%*CI* 1.161–258.859, *p* = 0.039). ROC analysis revealed the significant discriminative power of serum C1q levels for poor prognosis (AUC 0.781; 95%*CI* 0.673–0.888; *p* < 0.001) and DCI occurrence (AUC 0.763; 95%*CI* 0.637–0.888; *p* < 0.001). Higher C1q levels independently predicted a poor prognosis and DCI with equivalent predictive abilities to Hunt-Hess grade and modified Fisher grade (both *p* < 0.05).

**Conclusion:**

High levels of C1q in the blood is an independent risk factor for poor prognosis and the development of DCI in patients with aSAH. This can more objectively and accurately predict functional outcomes and the incidence of DCI. C1q may have a significant role in the mechanism behind DCI after aSAH.

## Introduction

Aneurysmal subarachnoid hemorrhage (aSAH) is a type of cerebrovascular disease that occurs when an aneurysm ruptures and blood enters the subarachnoid space. This accounts for 85% of all spontaneous subarachnoid hemorrhages (1, 2). The global incidence varies significantly (2), with lower rates reported in China due to factors such as high pre-hospital mortality rates (3). Risk factors for intracranial aneurysm rupture include advanced age, smoking, alcohol consumption, hypertension, diabetes and others (4). Symptoms of aSAH typically include sudden severe headache accompanied by nausea, vomiting, neck stiffness, neurological paralysis or epilepsy. Currently, digital subtraction angiography (DSA) is considered the gold standard for diagnosing aSAH (4). Rebleeding of intracranial aneurysms and cerebral vasospasm (CVS) are major prognostic factors affecting outcomes (3). The risk of rebleeding after aSAH is very high with extremely poor prognosis; therefore early surgical treatment should be performed to prevent rebleeding. Treatment methods may include endovascular intervention therapy or craniotomy clipping.

In recent years, advancements in preoperative management and surgical techniques for aneurysms have significantly reduced the incidence of re-rupture bleeding. However, DCI remains a severe complication of aSAH. Approximately 30% of patients experience DCI within 3–14 days after aSAH (5, 6). Traditionally, CVS was considered an important cause of DCI, typically occurring between days 4 and 14 after aneurysm rupture and spontaneously resolving after 21 days (7). Currently, nimodipine is the only drug proven to reduce the risk of DCI occurrence and improve outcomes post-aSAH due to its ability to prevent CVS (6, 8). The mechanism underlying the occurrence of DCI remains unclear to date, some scholars suggesting its potential association with neuroinflammatory responses (9). C1q plays an important role in inflammatory responses (10, 11) and is upregulated following acute brain injury (12–15). Its level is positively correlated with severity of neural damage and infarct area size (16, 17). Therefore, this study aims to analyze the relationship between serum C1q levels and functional prognosis in aSAH patients. This analysis will explore the pathophysiological mechanisms involving complement C1q in DCI. Ultimately, these findings could provide new insights into potential targets for drug therapy research.

## Materials and methods

### Study participants

The prospective cohort for this study comprised of patients diagnosed with aSAH in the Department of Neurosurgery at the First People’s Hospital of Hangzhou from January 2020 to October 2022, with a healthy control group also being selected. The inclusion criteria were as follows: (1) Diagnosed with subarachnoid hemorrhage via cranial CT scan, with cranial arterial aneurysms confirmed by computerized tomography angiography (CTA) or/and DSA; (2) Admitted within 24 h of symptom onset and underwent aneurysm clipping or embolization within 48 h of admission; and (3) Initial blood samples were collected within 24 h of admission after the first hemorrhage for laboratory testing. The exclusion criteria were as follows: (1) Under 18 years of age; (2) Aneurysm rebleeds before surgery; (3) Hemorrhage due to other causes; (4) Infectious inflammation; (5) History of autoimmune diseases; (6) Presence of other systemic diseases; (7) Prior cranial lesions; and (8) Lost to follow-up.

This study had obtained the approval of the Ethics Committee (Approval No. IIT-20230215-0023-02), and all research subjects or their legal representatives had signed informed consent forms.

### Clinical data collection

Patient histories and vital signs upon admission were collected from the hospital’s electronic medical record system. This included age, gender, history of hypertension, diabetes, smoking and alcohol consumption habits, as well as treatment details during hospitalization.

Upon patient admission and at the time of enrollment of control subjects, blood samples were collected from the peripheral veins of the forearm and placed into additive-free collection tubes. The samples were used to test white blood cell count, blood glucose levels and hemoglobin levels. Within 30 min, residual blood samples were centrifuged at 3000 *g* for 10 min, and the serum samples located in the upper layer were subsequently stored at −80°C for final analysis.

According to the manufacturer’s instructions, serum C1q levels were quantitatively determined using a commercially available assay kit (Shanghai Kexing Trading Co., Ltd., Shanghai, China) employing the enzyme-linked immunosorbent assay (ELISA) method. Every 3 months, two quantitative measurements were conducted by the same technician who was blind to the clinical data, and the average of the two measurements was used for the final analysis.

The location and size of the intracranial aneurysm were determined based on CTA or DSA. In selecting study parameters, we employed clinically recognized grading systems widely accepted in the field, specifically the Hunt-Hess grade system (Table 1) and modified Fisher grade system (Table 2) to assess the severity of patients upon admission with aSAH. Subsequently, patients diagnosed with aSAH were followed up for 3 months, and their neurological function status was comprehensively assessed based on the modified Rankin Scale (mRS) (Table 3). An mRS score of less than 3 indicated a good prognosis, while a mRS score of 3 or higher indicated a poor prognosis.

**Table 1:** Hunt-Hess grade in patients with subarachnoid hemorrhage due to aneurysm.

Grade	Description
Grade 0	Unruptured cerebral aneurysm
Grade I	Asymptomatic or minimal headache and slight nuchal rigidity
Grade II	Moderate to severe headache, nuchal rigidity, no neurologic deficit other than cranial nerve palsy
Grade III	Drowsy, minimal neurologic deficit
Grade IV	Stuporous, moderate to severe hemiparesis, possible early decerebrate rigidity, and vegetative disturbances
Grade V	Deep coma, decerebrate rigidity, moribund

**Table 2:** Modified Fisher grade of head CT scan in patients with subarachnoid hemorrhage caused by intracranial aneurysm.

Grade	Description
Grade 0	No bleeding or only intraventricular hemorrhage or intraparenchymal hemorrhage observed
Grade I	Only basal ganglia hemorrhage is observed
Grade II	Only observe hemorrhage in the surrounding ventricles or sulci
Grade III	Subarachnoid hemorrhage with intracerebral hematoma.
Grade IV	Thick intracerebral hemorrhage in the basal cisterns, periventricular spaces, and sulci.

**Table 3:** The modified Rankin Scale.

Scale	Symptoms
0	No symptoms at all
1	No significant disability. Able to carry out all usual activities, despite some symptoms
2	Slight disability. Able to look after own affairs without assistance, but unable to carry out all previous activities
3	Moderate disability. Requires some help, but able to walk unassisted
4	Moderately severe disability. Unable to attend to own bodily needs without assistance, and unable to walk unassisted
5	Severe disability. Requires constant nursing care and attention, bedridden, incontinent
6	Dead

### Treatment methods

In line with relevant aneurysm treatment guidelines (2, 3, 18–21), we treated patients with aSAH via craniotomy for aneurysm clipping or endovascular embolization. Following aneurysm occlusion, patients were managed according to standardized treatment guidelines for aSAH. Postoperatively, cranial CT scans were dynamically reviewed based on changes in the patient’s condition, to promptly detect iatrogenic cerebral infarction, brain edema, intracerebral hemorrhage and hydrocephalus. Patients were evaluated for the occurrence of DCI. The diagnostic criteria for DCI (22) were as follows: (1) Clinical examination revealed new focal neurological deficits (such as hemiplegia, hemianopia, aphasia, apraxia) and consciousness impairment; (2) A decrease of 2 or more points on the Glasgow Coma Scale (GCS) (Table 4), which could not be attributed to other causes based on laboratory test results, cranial imaging examination, and clinical assessment. At least one of the above criteria had to be met.

**Table 4:** Glasgow Coma Scale.

Scale	Eye opening response	Verbal response	Motor response
1	No response	No response	No response
2	To pain	Incomprehensible sounds	Extensor response, i.e., decerebrate posturing
3	To voice	Inappropriate words and jumbled phrases consisting of words	Abnormal flexion, i.e., decorticate posturing
4	Spontaneous	Confused, but able to converse	Withdraws from pain
5		Oriented	Localizes to pain
6			Obeys commands

If eye opening is not possible due to periorbital edema or fracture, “C” is used to denote the eye response; if normal verbalization is hindered due to tracheal intubation or tracheostomy, “T” is used to represent the verbal response. If there is a prior history of language impairment, “D” is used to denote the verbal response.

### Statistical analysis

We used SPSS 27.0 software (IBM Corp., Armonk, NY, USA) for statistical analysis and GraphPad Prism 8.0.2 version (GraphPad Software Inc., La Jolla, CA, USA) for data visualization. Categorical variables were presented as numbers and percentages and were analyzed using chi-square tests or Fisher’s exact tests. The Kolmogorov–Smirnov test was conducted to assess the normal distribution of quantitative variables. Continuous variables with normal and non-normal distributions were expressed as mean ± standard deviation and median (interquartile range), respectively. The correlation between serum C1q levels and patient Hunt-Hess grade, modified Fisher grade, and mRS score were analyzed using Spearman’s rank correlation test. Differences in serum C1q levels among different grade/score groups were compared using the Kruskal-Wallis rank sum test. Independent sample *t*-tests or Mann–Whitney U tests were employed for analyses, and variables with a *p* < 0.05 in univariate analysis were included in the Logistic regression model to explore the independent impact of serum C1q levels on functional prognosis and the occurrence of DCI. The receiver operating characteristic (ROC) curve analysis was used to assess the predictive ability of serum C1q levels for neurological function prognosis and the occurrence of DCI, and the reliability of predicting the prognosis of aSAH patients and the occurrence of DCI was evaluated based on the area under the curve (AUC). *p* < 0.05 was considered statistically significant.

## Results

### General clinical data of aSAH patients

**Figure 1:**
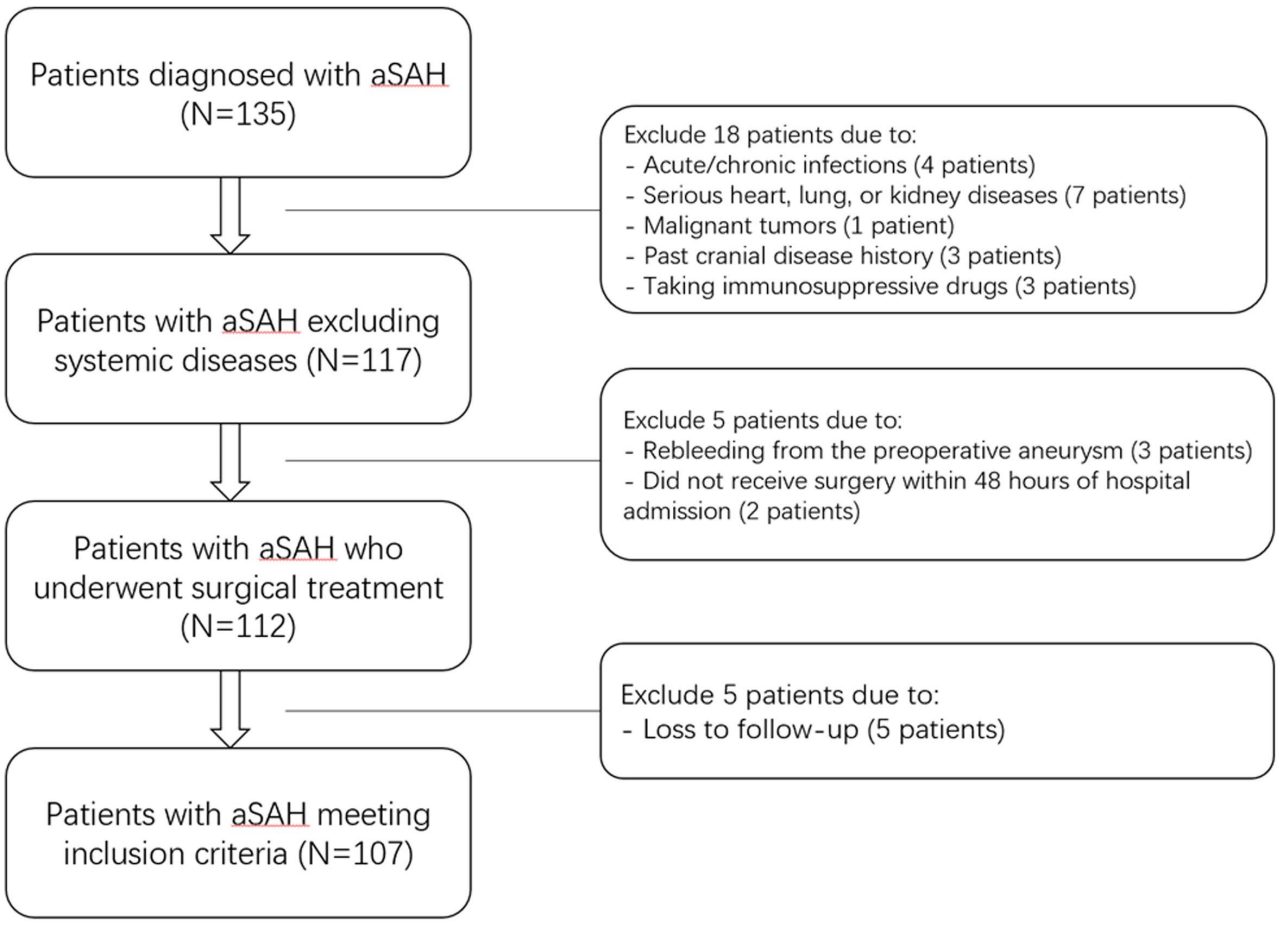
Flowchart of inclusion and exclusion criteria for patients with aneurysmal subarachnoid hemorrhage. Initially, 135 aneurysmal subarachnoid hemorrhage patients were assessed; thereafter, we excluded 28 patients; and ultimately, 107 patients were recruited. aSAH indicates aneurysmal subarachnoid hemorrhage.

Between January 2020 and October 2022, our hospital treated 135 patients diagnosed with aSAH. According to the exclusion criteria, 28 patients were excluded (Figure 1), leaving 107 patients in the case group for the study. Additionally, 107 healthy individuals were recruited as a control group. The general information, clinical indicators, radiological features and laboratory data for the 107 aSAH patients were presented in Table 5. The control group ranged in age from 32 to 88 years, with an average age of 58.68 ± 14.49 years; it comprised 46 males and 61 females, with 23 individuals (21.50%) having a history of alcohol consumption and 25 individuals (23.36%) having a history of smoking. Compared to the case group, there were no statistically significant differences in age, gender, history of alcohol consumption, or smoking history in the control group (all *p* > 0.05).

**Table 5:** General information, clinical indicators, radiological characteristics, and laboratory data of patients with aSAH.

Components	Mean ± Standard Deviation / Median (Interquartile Range) / Count (Percentage)	Range
Age (years)	57.16 ± 10.19	36–81
Sex (male/female)	49/58	
Hypertension	29 (27.1%)	
Diabetes	11 (10.3%)	
Alcohol consumption	30 (28.0%)	
Smoking	32 (29.9%)	
Hunt-Hess grade	2 (2–3)	1–5
Modified Fisher grade	2 (1–3)	1–4
Location (Anterior/Posterior circulation)	89/18	
Size (<5 mm/5-10 mm/>10 mm)	62/33/12	
Surgical Method (Embolization/Clipping)	79/28	
Systolic blood pressure (mmHg)	144.17 ± 20.80	87–201
Diastolic blood pressure (mmHg)	83.93 ± 11.48	51–119
Platelet count (10^9/L)	203.90 ± 58.29	64–337
Hemoglobin level (g/L)	134.27 ± 19.03	90–173
White blood cell count (10^9/L)	11.59 ± 3.81	4.90–23.50
Blood glucose level (mmol/L)	9.03 ± 1.92	3.56–15.42
Blood fibrinogen level (g/L)	2.84 ± 0.56	1.66–4.25
Blood D-dimer (mg/L)	1.05 (0.53–1.50)	0.06–6.00
Delayed cerebral ischemia (Present/Absent)	28/79	
Modified Rankin Scale	1 (0–3)	0–6
0	27	
1	27	
2	17	
3	11	
4	11	
5	7	
6	7	
Clinical outcome (Poor/Good)	36/71	

Quantitative data were reported as medians with 25th–75th percentiles or the mean ± standard deviation as appropriate. Qualitative data were presented as counts (proportions). Intergroup comparisons of various variables were performed using the *x*^2^ test or Fisher’s exact test for qualitative data, and Mann–Whitney *U*-test for quantitative data. aSAH indicates aneurysmal subarachnoid hemorrhage.

### Changes in serum C1q levels in aSAH patients

**Figure 2:**
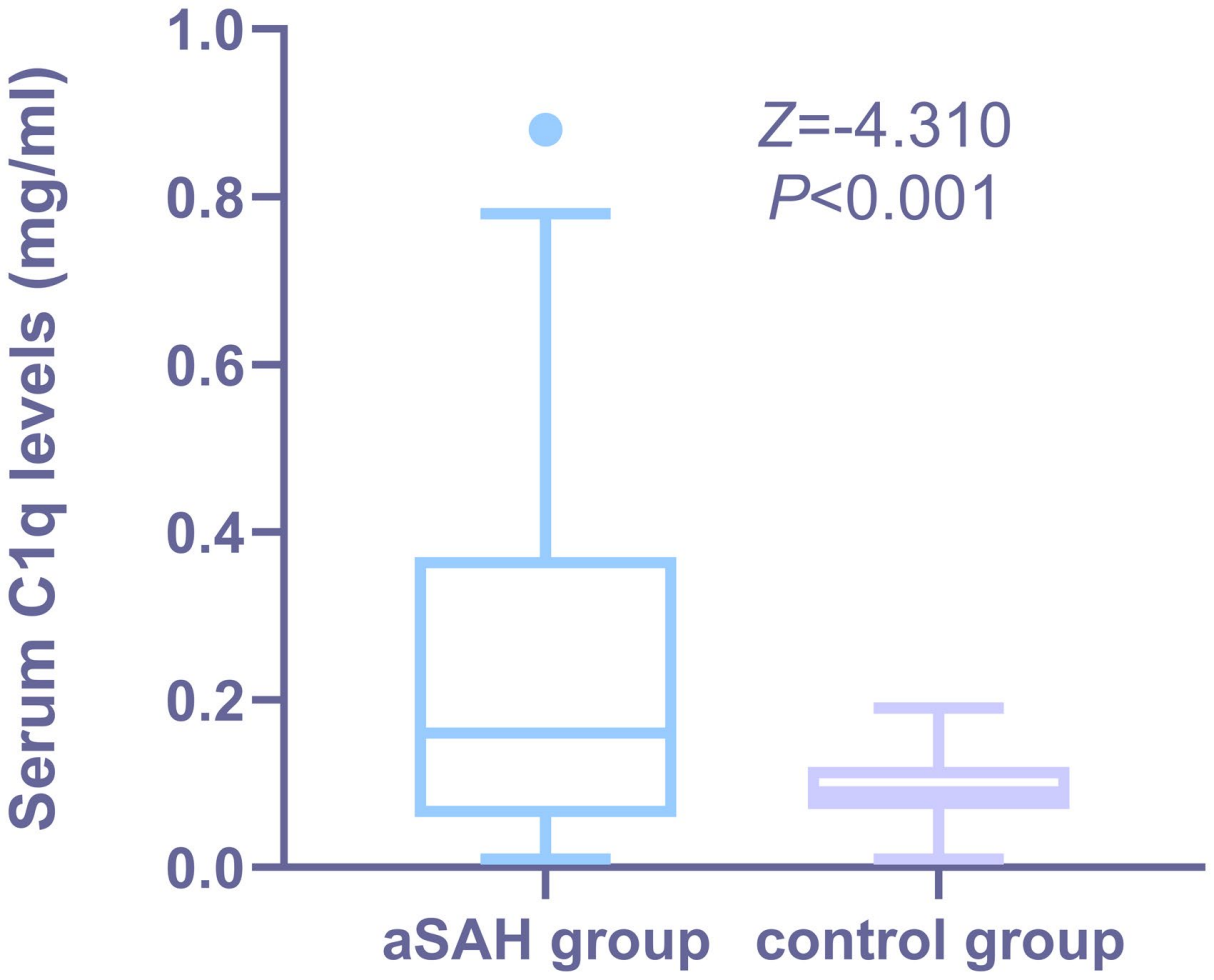
Comparison of serum C1q levels between patients with aSAH and healthy Individuals. Serum C1q levels were reported as median (upper-lower quartiles). Serum C1q levels were significantly higher in aSAH group than in control group using the Mann–Whitney U test (*P* < 0.001). The control group consisted of healthy individuals.

As shown in Figure 2, the serum C1q levels in aSAH patients was significantly higher than that in the control group, and the difference was statistically significant (*Z* = −4.310, *p* < 0.001).

### Univariate and multivariate correlation analysis of serum C1q levels with severity in aSAH patients

**Figure 3:**
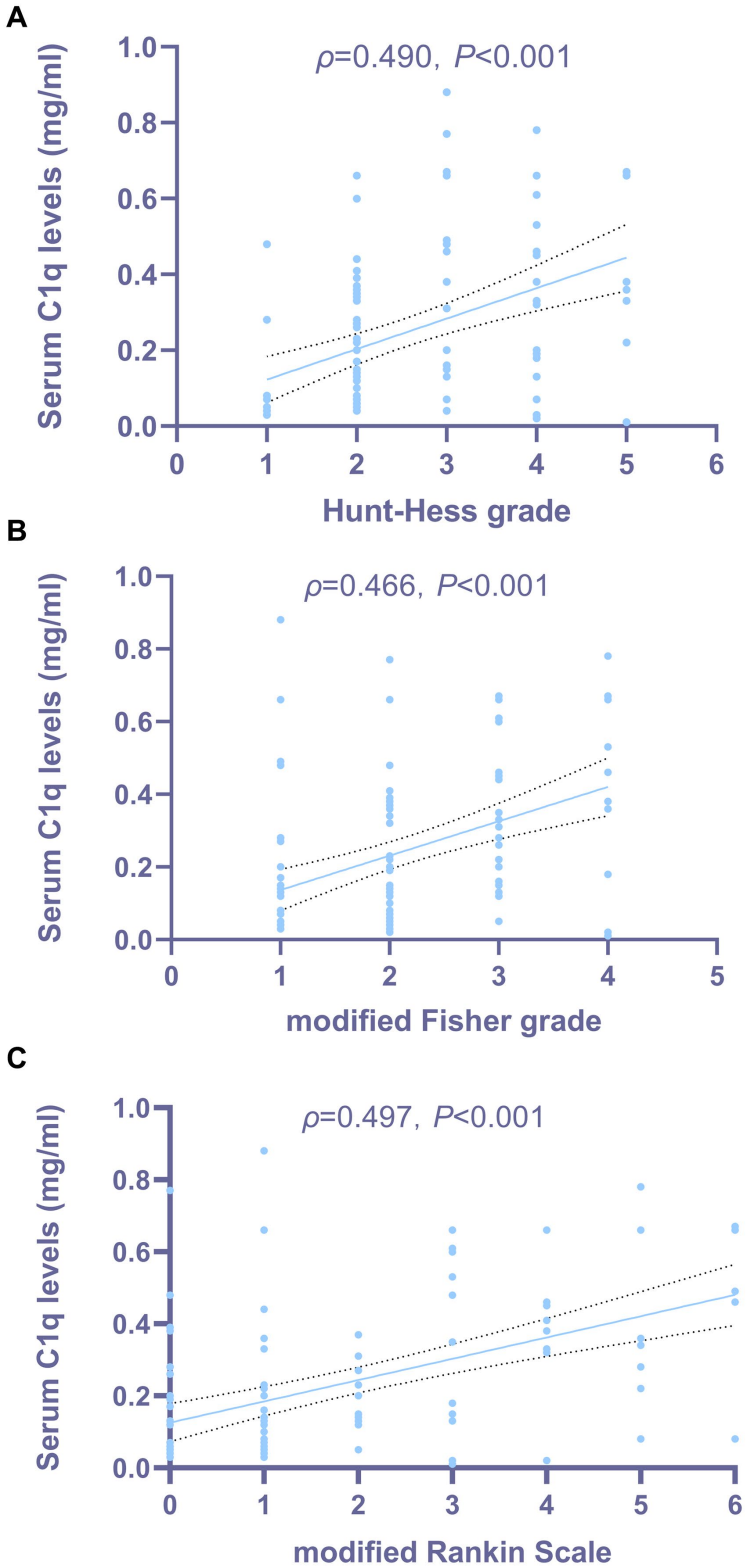
Relationship between serum C1q levels and the severity of aneurysmal subarachnoid hemorrhage. **(A)** Correlation analysis between serum C1q levels and Hunt-Hess grade in patients with aSAH. Serum C1q levels exhibited a strong correlation with the Hunt-Hess grade after aneurysmal subarachnoid hemorrhage, as indicated by Spearman’s correlation coefficient (*P* < 0.001). **(B)** Correlation analysis between serum C1q levels and modified Fisher grade in patients with aSAH. Serum C1q levels exhibited a strong correlation with the modified Fisher grade after aneurysmal subarachnoid hemorrhage, as indicated by Spearman’s correlation coefficient (*P* < 0.001). **(C)** Correlation analysis between serum C1q levels and modified Rankin Scale in patients with aSAH. Serum C1q levels exhibited a strong correlation with the modified Rankin Scale after aneurysmal subarachnoid hemorrhage, as indicated by Spearman’s correlation coefficient (*P* < 0.001).

**Figure 4:**
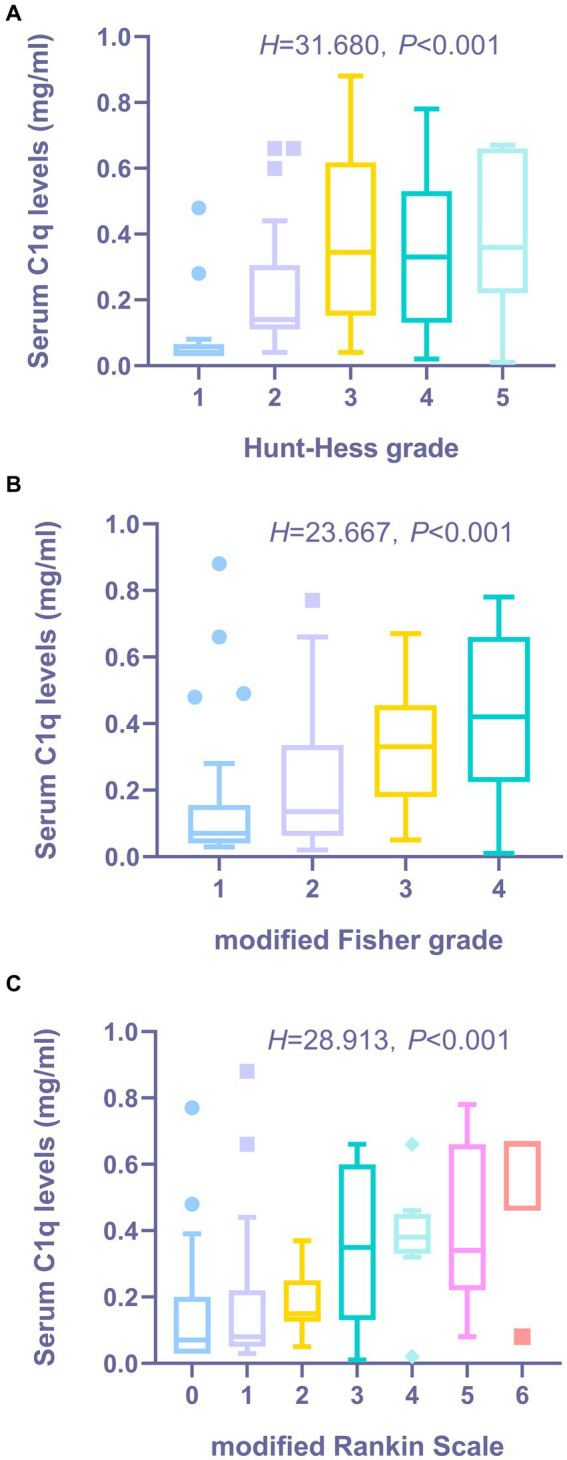
The distribution and comparative analysis of serum C1q levels in aSAH patients across various clinical grade/score groups. **(A)** Distribution and comparison of serum C1q levels in patients with aSHA across Hunt-Hess Grade Groups. The Kruskal-Wallis test revealed that the serum C1q levels post-aneurysmal rupture exhibited statistically significant differences across multiple groups (*P* < 0.001). **(B)** Distribution and comparison of serum C1q levels in patients with aSAH across modified Fisher grade groups. The Kruskal-Wallis test revealed that the serum C1q levels post-aneurysmal rupture exhibited statistically significant differences across multiple groups (*P* < 0.001). **(C)** Distribution and comparison of serum C1q levels in patients with aSAH across modified Rankin Scale groups. The Kruskal-Wallis test revealed that the serum C1q levels post-aneurysmal rupture exhibited statistically significant differences across multiple groups (*P* < 0.001).

Figures 3A,B show that the serum C1q levels had a significant positive correlation with the Hunt-Hess grade and modified Fisher grade (both *p* < 0.001). Table 6 shows that the serum C1q levels were also significantly correlated with the white blood cell count and blood glucose levels (both *p* < 0.05). Including these four variables in the multivariate model, Table 7 shows that the serum C1q levels were independently associated with the Hunt-Hess grade and modified Fisher grade (both *p* < 0.001). In addition, subgroup comparisons of serum C1q levels according to the Hunt-Hess grade and modified Fisher grade were conducted. The Kruskal-Wallis rank sum test revealed significant differences between groups (*H* = 31.680, *p* < 0.001; *H* = 23.667, *p* < 0.001). As shown in Figures 4A,B, with the increase in Hunt-Hess grade and modified Fisher grade, the serum C1q levels gradually increased.

**Table 6:** Correlation analysis of serum C1q levels in patients with aSAH.

Variable	*ρ*	*P*
Age (years)	0.106	0.276
Sex (male/female)	−0.029	0.768
Hypertension	0.120	0.219
Diabetes	0.015	0.874
Alcohol consumption	0.019	0.844
Smoking	0.079	0.417
Hunt-Hess grade	0.490	^**^ < 0.001
Modified Fisher grade	0.466	^**^ < 0.001
Modified Rankin Scale	0.497	^**^ < 0.001
Location (Anterior/Posterior circulation)	−0.081	0.405
Size (<5 mm/5–10 mm/>10 mm)	0.065	0.507
Surgical Method (Embolization/Clipping)	−0.027	0.781
Systolic blood pressure (mmHg)	0.069	0.480
Diastolic blood pressure (mmHg)	0.019	0.844
Platelet count (10^9/L)	0.124	0.202
Hemoglobin level (g/L)	0.002	0.986
White blood cell count (10^9/L)	0.314	^**^ < 0.001
Blood glucose level (mmol/L)	0.199	^*^0.040
Blood fibrinogen level (g/L)	−0.023	0.815
Blood D-dimer (mg/L)	0.175	0.071

Correlations were done using Spearman Correlation Coefficient in aneurysmal subarachnoid hemorrhage. C1q indicates complement component 1q. The asterisk indicates statistical significance (^*^p < 0.05).

**Table 7:** Multiple linear regression analysis of factors related to serum C1q levels in patients with aSAH.

Variable	*β* (95%*CI*)	VIF	*t*	*p*
Hunt-Hess grade	0.053 (0.015–0.091)	1.519	2.758	*0.007
Modified Fisher grade	0.049 (0.004–0.095)	1.593	2.136	*0.035
White blood cell count (10^9/L)	0.007 (−0.002–0.017)	1.048	1.516	0.133
Blood glucose levels (mmol/L)	0.012 (−0.007–0.031)	1.020	1.288	0.201

Correlations was presented using multivariate linear regression model. The asterisk indicates statistical significance (^*^p < 0.05).

### Univariate analysis and logistic regression analysis of factors affecting functional prognosis in aSAH patients

The Spearman rank correlation test was used to analyze the relationship between serum C1q levels and the 3-month mRS score of patients. As shown in Figure 3C, when the mRS score was considered as a continuous variable, there was a significant positive correlation between the serum C1q levels and the mRS score 3 months later (*ρ* = 0.497, *p* < 0.001). The mRS score 3 months later was grouped by grading, and the Kruskal-Wallis rank sum test was used to compare the differences in serum C1q levels distribution among mRS score groups. The results indicated: there was a statistically significant difference in the distribution of serum C1q levels among mRS score groups (*H* = 28.913, *p* < 0.001). It was observed that as the mRS score increased, the serum C1q levels significantly rose (Figure 4C).

**Figure 5:**
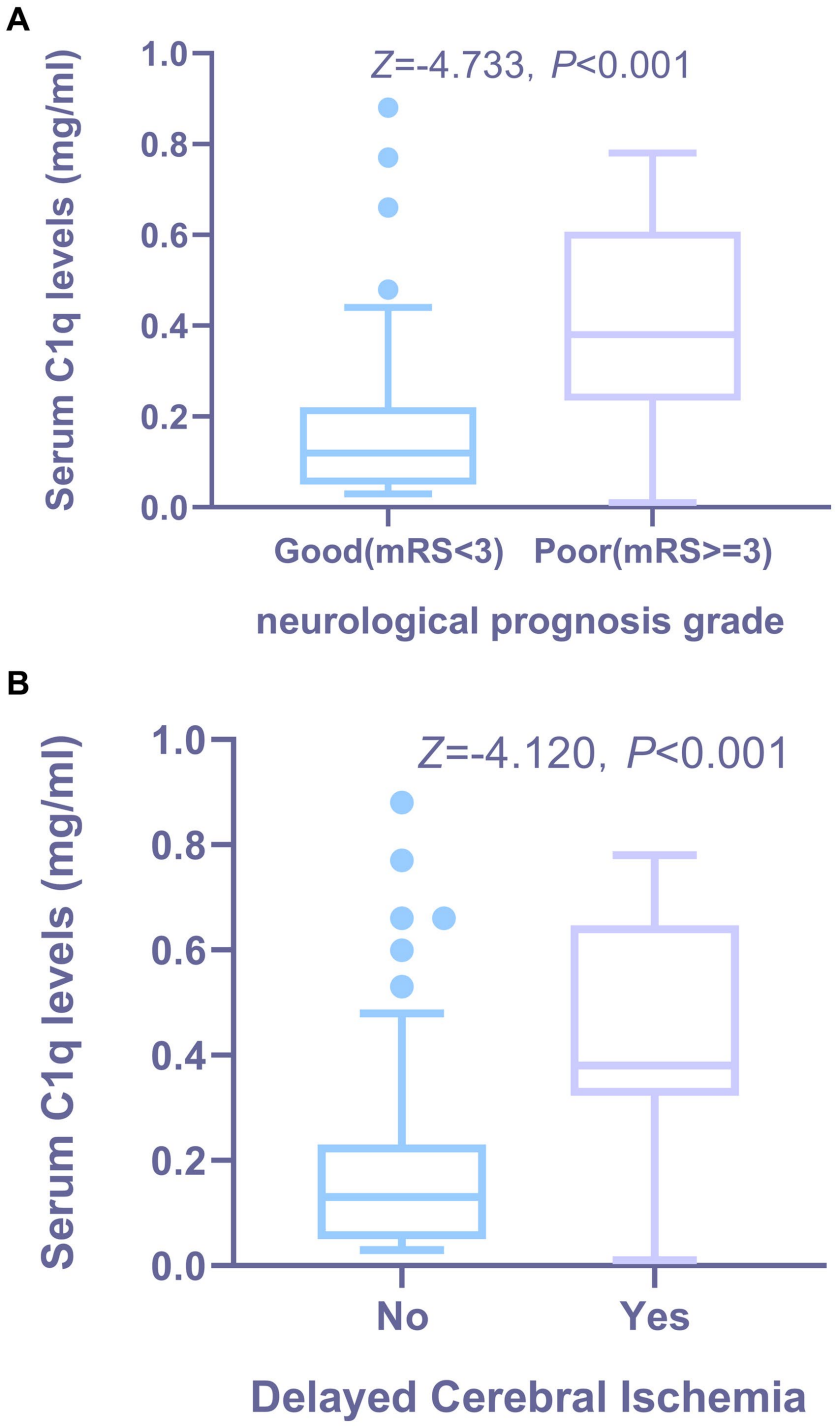
The relationship between serum C1q levels and functional prognosis, as well as the occurrence of DCI in patients with aSAH. **(A)** Distribution and comparison of serum C1q levels in patients with aSAH across neurological prognosis grade groups. Using the Mann-Whitney U test, the group with poor prognosis showed significantly higher serum C1q levels than the group with good prognosis (*P* < 0.001). **(B)** Distribution and comparison of serum C1q levels between patients with DCI and others following aSAH. Using the Mann-Whitney U test, patients who developed DCI had significantly higher serum C1q levels compared to those who did not develop DCI (*P* < 0.001). DCI indicates delayed cerebral ischemia.

36 patients had poor neurological prognosis. As shown in Figure 5A, the serum C1q levels in patients with poor prognosis was significantly higher than that in patients with good prognosis (*Z* = −4.733, *p* < 0.001). Further comparison of the differences in variables such as general information, clinical indicators, imaging features, and laboratory data between the two groups found that there were also significant differences in the Hunt-Hess grade, modified Fisher grade and hypertension at admission between the two groups (all *p* < 0.05), as shown in Table 8. After including the Hunt-Hess grade, modified Fisher grade, hypertension and serum C1q levels at admission in the Logistic regression equation, it was found that the Hunt-Hess grade, modified Fisher grade and serum C1q levels at admission were independently associated with poor prognosis in aSAH patients (*OR* = 2.924, *p* = 0.002; *OR* = 2.794, *p* = 0.009; *OR* = 36.927, *p* = 0.015), as shown in Table 9.

**Table 8:** Comparison of general information, clinical indicators, imaging features, and laboratory data between patients with different prognoses.

Variable	Good prognosis group (*n* = 71)	Poor prognosis group (*n* = 36)	*t*/*Z*/*χ^2^*	*p*
Age (years)	56.58 ± 9.54	58.31 ± 11.41	0.828	0.410
Sex (male/female)	31/40	18/18	0.387	0.534
Hypertension	14 (19.7%)	15 (41.7%)	5.824	^*^0.016
Diabetes	6 (8.5%)	5 (13.9%)	0.290	0.590
Alcohol consumption	18 (25.4%)	12 (33.3%)	0.754	0.385
Smoking	19 (26.8%)	13 (36.1%)	0.996	0.318
Hunt-Hess grade	2 (1–2)	4 (3–4)	−6.335	^**^ < 0.001
Modified Fisher grade	2 (1–2)	3 (2–4)	−5.832	^**^ < 0.001
Location (Anterior/Posterior circulation)	59/12	30/6	0.001	0.976
Size (<5 mm/5–10 mm/>10 mm)	44/20/7	18/13/5	1.425	0.490
Surgical Method (Embolization/Clipping)	54/17	25/11	0.541	0.462
Systolic blood pressure (mmHg)	142.72 ± 20.98	147.03 ± 20.43	1.013	0.313
Diastolic blood pressure (mmHg)	84.63 ± 10.86	82.53 ± 12.66	−0.896	0.372
Platelet count (10^9/L)	201.10 ± 55.61	209.42 ± 63.70	0.696	0.488
Hemoglobin level (g/L)	134.42 ± 18.75	133.97 ± 19.84	−0.115	0.909
White blood cell count (10^9/L)	11.20 ± 3.75	12.37 ± 3.86	1.508	0.134
Blood glucose level (mmol/L)	9.03 ± 1.70	9.03 ± 2.32	−0.010	0.992
Blood fibrinogen level (g/L)	2.82 ± 0.57	2.87 ± 0.54	0.416	0.679
Blood D-dimer (mg/L)	1.05 (0.47–1.41)	1.02 (0.70–1.77)	−0.844	0.399
Serum C1q level (mg/ml)	0.12 (0.05–0.22)	0.38 (0.24–0.61)	−4.733	^**^ < 0.001

Quantitative data were reported as medians with 25th–75th percentiles or the mean ± standard deviation as appropriate. Qualitative data were presented as counts (proportions). Intergroup comparisons of various variables were performed using the *x*^2^ test or Fisher’s exact test for qualitative data, and Mann–Whitney *U*-test for quantitative data. The asterisk indicates statistical significance (^*^p < 0.05).

**Table 9:** Logistic regression analysis of factors influencing the poor prognosis of patients with aneurysmal subarachnoid hemorrhage.

Variable	*B*	SE	Wald	OR (95%*CI*)	*p*
Hunt-Hess grade	1.073	0.341	9.874	2.924 (1.497–5.709)	*0.002
Modified Fisher grade	1.028	0.394	6.800	2.794 (1.291–6.049)	*0.009
Serum C1q levels (mg/ml)	3.609	1.487	5.891	36.927 (2.003–680.711)	*0.015
Hypertension	0.618	0.672	0.844	1.855 (0.497–6.926)	0.358

Results were showed as odds ratio (95% confidence interval) according to the binary logistic regression analysis. The asterisk indicates statistical significance (^*^p < 0.05).

### Univariate analysis and logistic regression analysis of factors affecting DCI occurrence in aSAH patients

28 patients developed DCI. As can be seen from Figure 5B, the serum C1q levels in patients who developed DCI were significantly higher than in patients who did not develop DCI (*Z* = −4.120, *p* < 0.001). Further comparison of the differences in variables such as general information, clinical indicators, imaging features, and laboratory data between the two groups found that there were significant differences in the Hunt-Hess grade and modified Fisher grade at admission between the two groups (all *p* < 0.001), as shown in Table 10. After including the Hunt-Hess grade, modified Fisher grade and serum C1q levels at admission in the Logistic regression equation, it was found that the Hunt-Hess grade, modified Fisher grade and serum C1q levels were independently associated with the occurrence of DCI in patients (*OR* = 2.513, *p* = 0.003; *OR* = 2.259, *p* = 0.023; *OR* = 17.334, *p* = 0.039), as shown in Table 11.

**Table 10:** Comparison of general information, clinical indicators, imaging features, and laboratory data between patients with and without DCI.

Variable	No delayed cerebral ischemia group (*n* = 79)	Delayed cerebral ischemia group (*n* = 28)	*t*/*Z*/*χ*^2^	*p*
Age (years)	56.35 ± 9.71	59.43 ± 11.30	1.378	0.171
Sex (male/female)	36/43	13/15	0.006	0.938
Hypertension	18 (22.8%)	11 (39.3%)	2.849	0.091
Diabetes	7 (8.9%)	4 (14.3%)	0.203	0.653
Alcohol consumption	20 (25.3%)	10 (35.7%)	1.108	0.293
Smoking	21 (26.6%)	11 (39.3%)	1.591	0.207
Hunt-Hess grade	2 (1–3)	4 (3–4)	−5.617	^**^ < 0.001
Modified Fisher grade	2 (1–2)	3 (2–4)	−5.318	^**^ < 0.001
Location (Anterior/Posterior circulation)	67/12	22/6	0.216	0.642
Size (<5 mm/5–10 mm/>10 mm)	51/21/7	11/12/5	5.546	0.062
Surgical Method (Embolization/Clipping)	60/19	19/9	0.701	0.403
Systolic blood pressure (mmHg)	144.08 ± 21.46	144.43 ± 19.18	0.077	0.939
Diastolic blood pressure (mmHg)	84.78 ± 11.58	81.50 ± 11.04	−1.305	0.195
Platelet count (10^9/L)	200.03 ± 56.59	214.82 ± 62.60	1.156	0.250
Hemoglobin level (g/L)	135.84 ± 19.65	129.86 ± 16.69	−1.436	0.154
White blood cell count (10^9/L)	11.56 ± 3.92	11.67 ± 3.55	0.129	0.898
Blood glucose level (mmol/L)	9.00 ± 1.66	9.12 ± 2.55	0.236	0.814
Blood fibrinogen level (g/L)	2.84 ± 0.55	2.85 ± 0.58	0.083	0.934
Blood D-dimer (mg/L)	1.03 (0.47–1.41)	1.20 (0.68–1.79)	−1.226	0.220
Serum C1q level (mg/ml)	0.13 (0.05–0.23)	0.38 (0.32–0.65)	−4.120	^**^ < 0.001

Quantitative data were reported as medians with 25th–75th percentiles or the mean ± standard deviation as appropriate. Qualitative data were presented as counts (proportions). Intergroup comparisons of various variables were performed using the *x*^2^ test or Fisher’s exact test for qualitative data, and Mann–Whitney *U*-test for quantitative data. DCI indicates delayed cerebral ischemia. The asterisk indicates statistical significance (^*^p < 0.05).

**Table 11:** Logistic regression analysis of factors affecting delayed cerebral ischemia in patients with aneurysmal subarachnoid hemorrhage.

Variable	B	SE	Wald	OR (95%*CI*)	*p*
Hunt-Hess grade	0.921	0.308	8.926	2.513 (1.373–4.599)	*0.003
Modified Fisher grade	0.815	0.360	5.134	2.259 (1.116–4.571)	*0.023
Serum C1q levels (mg/ml)	2.853	1.379	4.277	17.334 (1.161–258.859)	*0.039

Results were showed as odds ratio (95% confidence interval) according to the binary logistic regression analysis. The asterisk indicates statistical significance (^*^p < 0.05).

### ROC curve analysis of serum C1q levels predicting functional prognosis and DCI occurrence in aSAH patients

**Figure 6:**
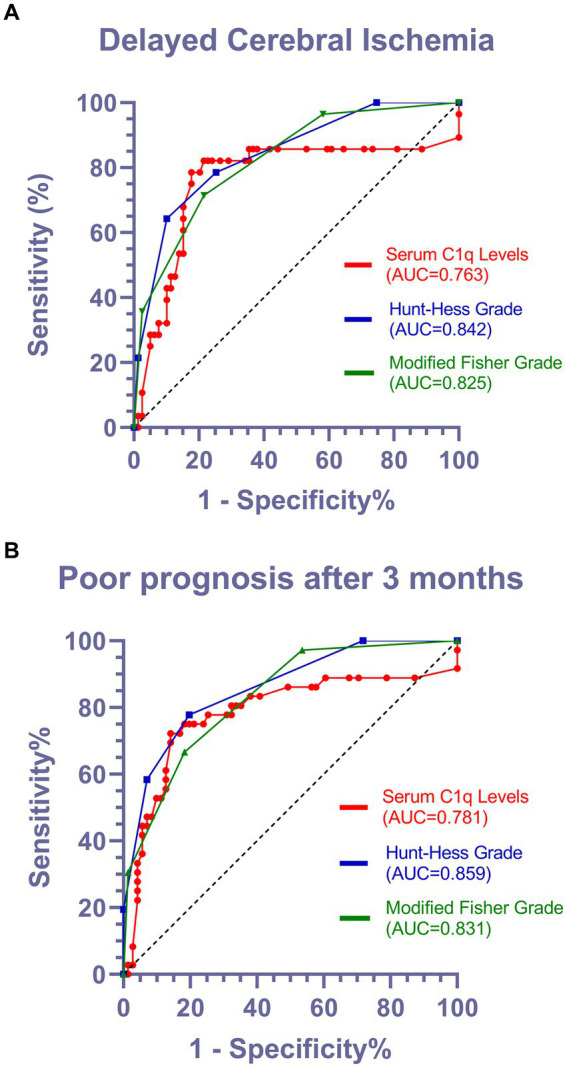
Predictive value of serum C1q levels for the occurrence of DCI and poor prognosis after 3 months in patients with aSAH. **(A)** Receiver operating characteristic of serum C1q levels in predicting the occurrence of DCI in patients with aSAH. Under the receiver operating characteristic curve, serum C1q levels significantly discriminated development of DCI (area under curve 0.763; 95% confidence interval, 0.637–0.888; *P* < 0.001). Using maximum Youden method, serum C1q level>0.315mg/ml distinguished patients at risk of DCI with 78.6% sensitivity and 82.3% specificity. AUC denotes area under curve. **(B)** Receiver operating characteristic curve for serum C1q levels in predicting the functional prognosis of patients with aSAH. According to the receiver operating characteristic curve, serum C1q levels significantly distinguishes the development of poor prognosis (area under the curve 0.781; 95% confidence interval, 0.673–0.888; *P* < 0.001). Using the maximum Youden method, a serum C1q level>0.315mg/ml can identify patients at risk of poor prognosis after three months, with a sensitivity of 72.2% and specificity of 85.9%. AUC represents the area under the curve.

Using ROC curve analysis, serum C1q levels could significantly predict the occurrence of DCI and poor prognosis at 3 months, and the corresponding serum C1q levels values were determined using the Youden method, predicting DCI and poor prognosis with medium to high specificity and sensitivity, as shown in Figures 6A,B.

## Discussion

As understanding and treatment of aSAH have become more standardized, despite a decrease in mortality, its high disability rate has significantly impacted patients’ families and society (16, 23). Many factors, such as neurological status upon admission, age and amount of bleeding, are closely related to the prognosis of aSAH patients. DCI approximately affects 30% of the patients and is generally believed to be associated with risk factors such as history of smoking and drinking, hyperglycemia, and early systemic inflammatory response, etc. (20). Recent studies have suggested that CVS, microthrombus formation, cortical spreading depolarization and neuroinflammation are all associated with and probably lead to DCI, which is the most feared complication (21).

Although there are currently some scoring scales to assess disease severity and predict prognosis (18, 19), they are limited by the subjectivity of clinicians and cannot objectively and accurately predict patients’ functional prognosis and the occurrence of DCI.

The complement system has been proven to play an important role in acute brain injury (9), ultimately leading to secondary brain injury and blood–brain barrier damage (24). Studies have shown that after cerebral hemorrhage, the complement activation participates in the formation of cerebral edema through the inflammatory response (25). Other research indicates that after acute brain injury, the blood–brain barrier is damaged, and the influx of inflammatory mediators, plasma proteins and complement proteins drives a delayed secondary inflammatory response (26). In general, an excessively activated complement system can lead to tissue and organ damage (27), which is not conducive to neurological recovery.

C1q is the initiator of the classical pathway of the complement cascade and participates in immune-inflammatory responses: activators bind to C1q, sequentially activate C1r, C1s, C4, C2 and C3, forming C3 convertase (C4b2a) and C5 convertase (C4b2a3b). C3 convertase cuts C3 into C3a and C3b, C3a can activate mast cells and macrophages, triggering an inflammatory response (28); C5 convertase cuts C5 into C5a and C5b, C5a can promote the development of the inflammatory response, while C5b can assemble with C6-C9 to form the membrane attack complex (MAC), causing cell lysis (29).

The study indicates that the levels of C1q in cerebrospinal fluid (CSF) significantly increase after cerebral ischemic injury, rising more than 5-fold compared to the control group at 24 h (15). Additionally, in traumatic brain injury, the immunoreactivity of C1q was measured using a computerized image system, revealing a significant elevation in C1q levels compared to the control group (*p* = 0.0073) (14). Consequently, marked increases in serum C1q levels were observed in both conditions. In contrast to the observed elevation in C1q levels post-injury, some researchers have found that C1q deficiency in neonatal mice demonstrates a neuroprotective effect. When compared to wild-type mice, those lacking the C1q gene exhibited significantly smaller brain infarct volumes and fewer neurofunctional deficit in the context of hypoxic–ischemic brain injury (30).

Interestingly, we also note the role of other biomarkers identified in brain injury research by some scholars, such as the increased expression of intracellular enzymes Nox2 and Nox4. They generate a large amount of reactive oxygen species (ROS) by reducing oxygen to superoxide anions. These ROS may disrupt the growth and activity of neurons, leading to damage in brain tissue. Additionally, Nox2 and Nox4 may also play a role in astrocytes, causing damage to the cells themselves or the surrounding neurons under stressful conditions. These effects may be associated with inflammation and neuronal cell death, further exacerbating the severity of brain injury (31). Another example is the neuroprotective role of the Cellular prion protein (PrPc) in ischemic injury. Firstly, PrPc, as a signaling partner of the PI3K/Akt and MAPK pathways, can regulate cell survival and proliferation. Secondly, as a modulator of glutamate receptors, PrPc is involved in the protection of neurons and synaptic plasticity. Moreover, PrPc promotes the homing mechanism of stem cells, leading to vascular generation and neurogenesis (32). These studies, akin to our findings on C1q, although varying in mechanisms of action, underscore the potential value of serological markers in disease prognosis.

In our study, the levels of C1q in the serum of aSAH patients were significantly higher than those in the healthy control group, and they were independently associated with Hunt-Hess grade, modified Fisher grade and mRS score. Also, the increase in C1q levels was significantly related to poor prognosis and the occurrence of DCI with a medium to high level of specificity and sensitivity. This may suggest that serum C1q levels could be a potential biomarker for the prognosis of aSAH.

In Figures 6A,B, it is observed that the Area Under the Curve (AUC) values for Hunt-Hess grade and modified Fisher grade were superior to those for C1q levels. In light of this, we acknowledge that Hunt-Hess grade and modified Fisher grade, serving as established and traditional scoring systems, indeed demonstrate high predictive value. However, it is noteworthy that both Hunt-Hess grade and modified Fisher grade necessitate clinical evaluation of patient status or meticulous review of CT scans, introducing subjectivity and placing a high demand on the professional expertise of physicians. In contrast, C1q, as a readily measurable and objective parameter, effectively mitigates these drawbacks. According to the Maximum Youden Index on the AUC curve, when serum C1q levels exceed 0.315 mg/mL, patients are more prone to developing DCI and exhibit a poor prognosis. This finding underscores the potential of C1q levels as a straightforward and objective indicator, serving as a robust predictor for both the occurrence of DCI and prognosis, while circumventing the inherent limitations associated with traditional scoring systems.

This study also has some limitations. Firstly, this study is a single-center prospective cohort study with a small sample size, and the conclusions need further validation from more prospective, multi-center, large-sample studies. Secondly, this study only collected and analyzed the serum C1q levels at admission, and with the progression and treatment of aSAH, the changes in serum C1q levels may show other trends. Monitoring and analyzing the serum C1q levels at each stage of aSAH progression could potentially reveal more valuable predictive information. Thirdly, regrettably, we did not conduct CSF analysis. Collection of CSF typically requires specialized medical procedures and may cause discomfort to patients. Some patients might even express concerns or reluctance toward CSF collection. Therefore, we opted for the collection of peripheral venous blood samples, a relatively less invasive procedure that is more routine, straightforward and easier to obtain patient cooperation.

## Conclusion

First, a high level of serum C1q may serve as an independent risk factor for poor prognosis and the occurrence of DCI in patients with aSAH. Second, serum C1q levels, as a simple and effective serum biomarker, may assist clinicians in more accurately predicting the functional prognosis and the occurrence of DCI in patients with aSAH.

## Data availability statement

The original contributions presented in the study are included in the article/Supplementary material, further inquiries can be directed to the corresponding author.

## Ethics statement

The studies involving humans were approved by the Ethics Committee of Hangzhou First People’s Hospital, Hangzhou First People’s Hospital. The studies were conducted in accordance with the local legislation and institutional requirements. The participants provided their written informed consent to participate in this study.

## Author contributions

LW: Writing – original draft. HZ: Writing – review & editing. WZ: Writing – original draft. HW: Data curation, Writing – review & editing. ZW: Data curation, Writing – review & editing. XD: Formal analysis, Supervision, Writing – review & editing. QD: Project administration, Resources, Supervision, Writing – review & editing.
