# Corrigendum: Clinical value of serum complement component 1q levels in the prognostic analysis of aneurysmal subarachnoid hemorrhage: a prospective cohort study

**DOI:** 10.3389/fneur.2024.1399344

**Published:** 2024-03-22

**Authors:** Linjie Wang, Haotian Zhou, Wenhao Zheng, Heng Wang, Zheng Wang, Xiaoqiao Dong, Quan Du

**Affiliations:** ^1^The Fourth School of Clinical Medicine, Zhejiang Chinese Medical University, Hangzhou, China; ^2^Department of Neurosurgery, Affiliated Hangzhou First People's Hospital, School of Medicine, Westlake University, Hangzhou, China

**Keywords:** aneurysmal subarachnoid Hemorrhage, Complement component 1q, prognosis, delayed cerebral ischemia, biomarker

In the published article, there was an error in affiliation(s) [1,2]. Instead of

^1^ The Fourth Clinical Medical College, Zhejiang Chinese Medical University, Hangzhou, China

^2^ Department of Neurosurgery, The First People's Hospital of Hangzhou, Zhejiang University School of Medicine, Hangzhou, China

It should be

^1^ The Fourth School of Clinical Medicine, Zhejiang Chinese Medical University, Hangzhou, China

^2^ Department of Neurosurgery, Affiliated Hangzhou First People's Hospital, School of Medicine, Westlake University, Hangzhou, China

The authors apologize for this error and state that this does not change the scientific conclusions of the article in any way. The original article has been updated.

